# Varicella seroprevalence in healthcare workers in a tertiary hospital: an audit of cross-sectional data

**DOI:** 10.1186/s13104-015-1656-0

**Published:** 2015-11-10

**Authors:** Alexander Wilhelm Gorny, Chikul Mittal, Sharon Saw, Indumathi Venkatachalam, Dale Andrew Fisher, Paul Anantharajah Tambyah

**Affiliations:** National University Hospital, 5 Lower Kent Ridge Road, Singapore, 119074 Republic of Singapore

**Keywords:** Healthcare worker, Seroprevalence, Varicella, Immunisation

## Abstract

**Background:**

The seroprevalence of varicella in Southeast Asia is not well described especially in healthcare workers (HCW) in the region. We report the varicella seroprevalence among healthcare workers from a diverse range of countries working in a tertiary care hospital in Singapore.

**Methods:**

We audited the results of annual HCW health screening, which included a varicella assay, from the years 2009 to 2014. During this period, there was a change in hospital policy mandating varicella immunity for all newly employed healthcare workers. The serological data were reviewed with employment records on occupation and nationality. Seroprevalence rates were determined by standard commercial enzyme linked immunosorbent assays for each year of testing. Odds of being immune in 2014 were compared by means of multiple logistic regression.

**Results:**

A total of 10,585 samples were obtained from 6668 unique individuals over four separate cross-sections of the hospital workforce. A peak seroprevalence of 92.8 % (95 % CI 92.0–93.5) was recorded in 2014. Younger employees had a lower seroprevalence than their older colleagues. In a consolidated sample of 4875 members of the active workforce in October 2014, we identified that Indian nationals were less likely to be immune than their Singaporean national colleagues, odds ratio (OR) 0.26 (95 % CI 0.17–0.43, p < 0.001), while Chinese nationals were more likely to be immune, OR 4.34 (95 % CI 1.61–12.2, p = 0.004), after controlling for year of screening, gender, age-group and vocation. In 2014, being employed as administrative staff, OR 0.43 (95 % CI 0.29–0.64, p < 0.001) or contract service provider, OR 0.30 (95 % CI 0.19–0.47, p < 0.001), was also associated with a lower odds of being immune than being employed as a nurse.

**Conclusions:**

There remain a small number of healthcare workers who are non-immune to varicella in our tertiary hospital. A new pre-employment policy of mandatory screening and vaccination may have increased rates of immunity but more needs to be done to ensure that all of our employees are immune to varicella to protect our vulnerable patients.

## Background

Hospitals need to monitor the susceptibility of their employees to varicella zoster virus (VZV) in order to address the risks of nosocomial and occupational transmissions among patients, staff and visitors [1]. Although the infections which healthcare workers acquire from patients are usually self-limited and minor, the consequences of nosocomial varicella infection in patients can be severe especially in infants and the immunocompromised [2].

Varicella vaccination has been available in Singapore since 1996 [3] but has not been mandated in the national immunization programme. This and a low rate of transmission are thought to have given rise to national seroprevalence rates of 34.5 % in children ages 1–6 years, 60.5 % in children 7–12 years and 71.0 % in ages 13–17 years as assessed between the years 2008 and 2010 [4]. Chickenpox had been a notifiable disease in Singapore until 2008, after which the legal requirement for reporting was removed [4]. Since 2009 our hospital has extended voluntary free varicella vaccination to all employees, clinical, administrative and service partners.

The National University Hospital (NUH) in Singapore employed 7349 full-time staff as of 31 October 2014 in addition to facilities management staff who are employed by external service partners. Every year in October the hospital has provided free cardiovascular screening to its employees, measuring among other parameters: body mass index, blood pressure, fasting cholesterol and glucose levels. A varicella Immunoglobulin G (IgG) assay was included from 2009 to establish a baseline immunity profile, other occupationally relevant assays, e.g. measles, mumps and rubella, were included in subsequent years. Employees who were found to be non-immune would later be invited for a voluntary consultation with the occupational health physician, who would review electronic medical records and help determine the need for vaccination—all at no additional cost. Mandatory serological assessment and vaccination previously only applied to staff working in high risk areas and depended on history of vaccination or clinical disease. A change in pre-employment policy effected on 01 July 2013 meant that all prospective employees were assessed for documented history of exposure and/or vaccination before using their serology to assess the need for vaccination. An audit into seroprevalence data generated through the annual health screening programme was undertaken in 2014 to better understand possible gaps in varicella immunisation coverage.

## Methods

Our audit was conducted under the direction of the hospital’s infection control committee and relied on the electronic records generated by the annual health screening programme from 2009 to 2014. Secondary analysis of this dataset was in keeping with national guidelines which stipulate that licensed healthcare establishments evaluate infection control procedures on a continuing basis [5]. These evaluations are governed by Singapore Ministry of Health regulations as stated in the Private Hospitals and Medical Clinics Act and are independent of our institution’s domain specific ethical review board.

All serum specimens dating back to 2009 had been tested using an anti-VZV enzyme-linked immunosorbent IgG assay (ELISA), *Euroimmun Medizinische Labordiagnostika AG*, Germany. The manufacturers’ insert reports 100 % sensitivity and specificity of their assay as proven on 265 clinically characterised patient samples. In our analysis we used the manufacturer-recommended upper limit of the reference range for non-immunity at 100 IU/L. This cut-off showed a 97.4 % seroprevalence when validated on 500 health blood-donor serum samples in Germany, reflecting the known percentage of infected adults. For the purposes of this audit we did not distinguish individuals falling into the ‘borderline’ range 80–109 IU/L as defined by the manufacturers or explore their status on follow-up. Test results were coded for each participating employee’s unique identification number, gender and date of birth. The results of each screening exercise were compiled using Microsoft Excel software. Information on vocation and nationality were joined to these data-points using human resource records current as of 31 Oct 2014 using Quantum Geographic Information System (GIS) [6].

Basic descriptive indicators for the data-set were generated using the summary and tabulation functions of STATA Version 13.1 (Stata Corp, College Station, TX, USA). We compared year-on-year seroprevalences by drawing binomial 95 % confidence intervals (CI) around point-estimates generated for each year of testing. The variable for age was categorised into decades of birth in order to account for changes in age across annual cross-sections. Thereafter, a seroprevalence curve was plotted to illustrate age-specific estimates in each year of the health screening programme.

Using individual employee data current as of October 2014 and the most recent serology result from all previous years of testing, we were able to review a more complete cross-section of our employees in 2014, and report crude seroprevalence estimates by nationality and vocation. The consolidated data-set was subjected to multiple logistic regression analysis comparing the odds of being immune across the following exposure variables: year result had been obtained, age, gender, nationality and vocation. A Strengthening the Reporting of Observational Studies in Epidemiology (STROBE) statement check-list [7] was used in compiling this report.

## Results

Over the four batches of screening involving a total of 6701 individuals, data on 33 (0.5 %) individuals had to be omitted in view of incomplete or erroneous data. Applying manufacturers’ definitions, 6134 (91.7 %) titres were positive, 106 (1.6 %) were borderline and 460 (6.9 %) fell into the negative range. In this audit we did not review follow-up results for ‘borderline’ individuals, and instead classified 89 individuals with titres 0–100 IU/L as ‘non-immune’ and 17 individuals with titres between 101 and 109 IU/L as ‘immune’.

VZV seroprevalence was not assessed in 2010 or 2013 in view of competing priorities during the annual staff health screening exercise. The majority of employees presented for screening more than once in all 4 years of screening (see Table 1), but the highest participation rate was registered in 2014 covering 44 % of full-time staff, owing to enhanced health promotion efforts to participate in health screening. The average age in all four sets of data ranged between 38 and 40 years. The age-group specific proportions of female staff ranged from 79 to 91 %.

**Table 1:** Characteristics of cross-sectional samples 2009–2014, n = 10,525 data-points

Year	Gender (n)	Decade of birth				Average age in years (s.d.)	Participated in one screening only (%)	Total
		≤1959	1960–1969	1970–1979	≥1980			
2009	Female (2023)	361	428	786	448	39 (11)	1030 (43)	2388
	Male (365)	98	70	132	65			
2011	Female (1885)	238	357	680	610	38 (10)	552 (25)	2199
	Male (314)	65	63	109	77			
2012	Female (2017)	219	343	666	789	38 (10)	688 (30)	2301
	Male (284)	54	48	101	81			
2014	Female (3071)	380	556	944	1191	40 (11)	1806 (50)	3637
	Male (566)	96	106	186	179			

**Fig. 1:**
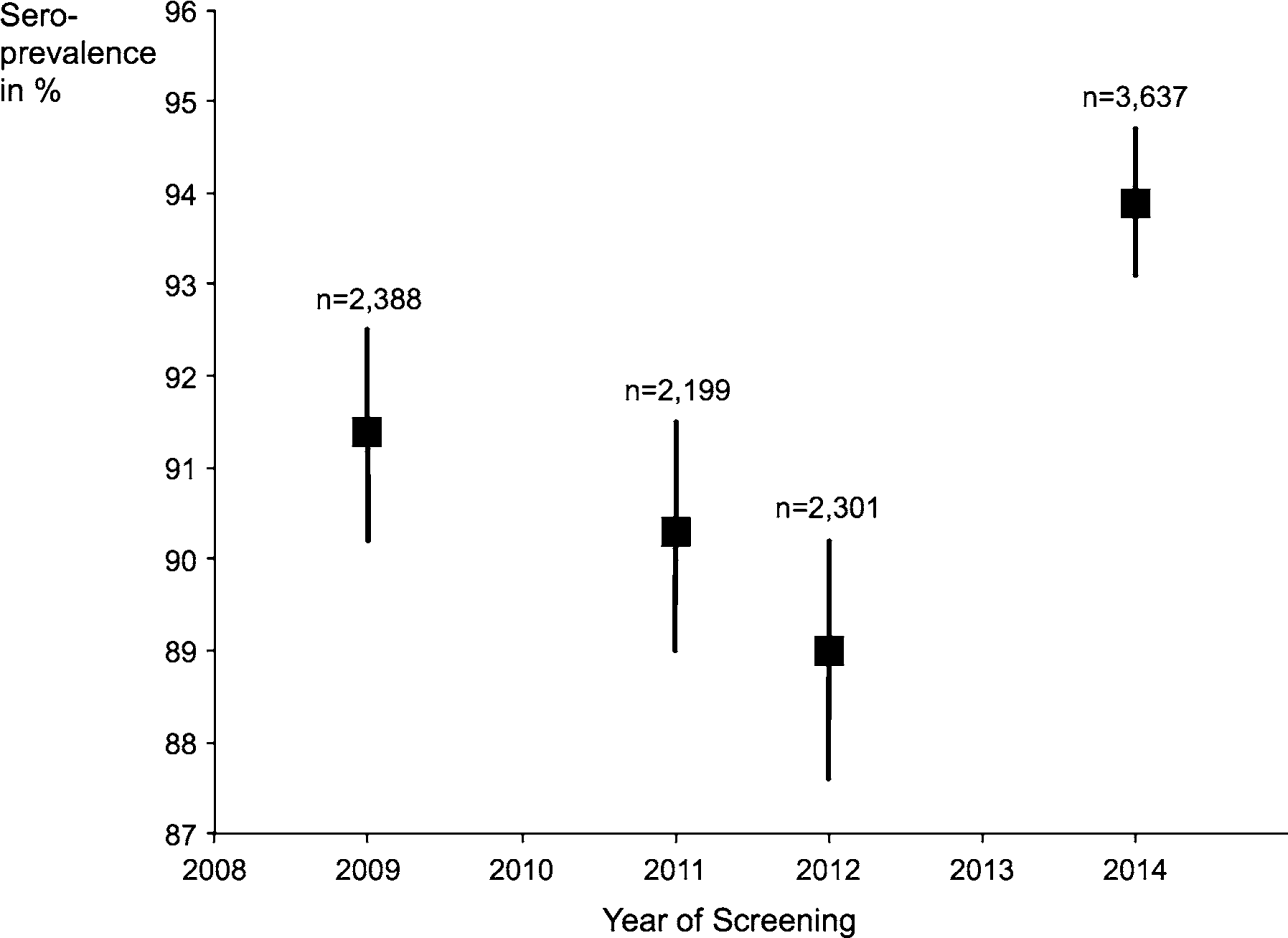
Crude seroprevalence by year of screening with 95 % confidence intervals. We calculated and plotted crude seroprevalence figures for each year of health screening. Error bars were computed using binomial 95 % confidence intervals for the proportions of employees found to be immune on varicella serology

**Fig. 2:**
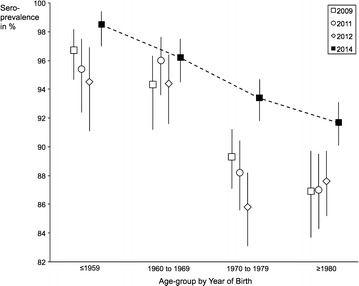
Age-group specific seroprevalence with 95 % confidence intervals. We have plotted age-group specific seroprevalence rates with error bars for the health screenings performed in (1) 2009, (2) 2011, (3) 2012 and (4) 2014. For the purpose of illustration we have added the seroprevalence curve for 2014

Crude point-estimates of varicella seroprevalence declined from 2009 through to 2012, followed by an increase to 93.9 % (95 % CI 93.1–94.7) in 2014—see Fig. 1. This development coincided with the change in pre-employment policy. The seroprevalence curves for each year were similar in shape across the different age groups—see Fig. 2.

For the most recent and most complete year of 2014, omitting erroneous data, we were able to identify the immunity status of 4464 members of staff by consolidating data from 2009 to 2014, thus representing 60.7 % (4464/7349) of the directly employed workforce. We compared the demographic characteristics of participating staff members against the whole hospital in Table 2. We counted 411 contracted service-provider staff in the 2014 sample. Adding service-providers into the 2014 sample of 4875 individuals, we calculated an overall seroprevalence of 92.8 % (95 % CI 92.1–93.5) which was not significantly lower than the 2014 point estimate.

**Table 2:** Characteristics of hospital employees in october 2014

	Participated in at least one screening since 2009 (% of total)	No health screening data (% of total)	Hospital total
Overall	4465 (60.4)	2929 (39.6)	7394
Gender			
Female	3928 (64.7)	2145 (35.3)	6073
Male	537 (40.7)	784 (59.4)	1321
Vocation			
Nursing	2221 (65.4)	1176 (34.6)	3397
Allied health	818 (61.5)	513 (38.5)	1331
Ancillary	744 (61.4)	467 (38.6)	1211
Doctor/dentist	124 (18.6)	543 (81.4)	667
Administrators	558 (70.8)	230 (29.2)	788
Age-group by year of birth			
≤1959	558 (68.7)	254 (31.3)	812
1960–1969	783 (65.6)	411 (34.4)	1194
1970–1979	1429 (60.5)	934 (39.5)	2363
≥1980	1695 (56.1)	1329 (44.0)	3024

We analysed the seropositivity for the different vocations and nationalities, generating crude seroprevalence estimates with 95 % CIs. The results are shown in Tables 3 and 4.

**Table 3:** Crude seroprevalence by vocation in 2014 (n = 4875)

Vocation	n	Seroprevalence in % (binomial 95 % CI)
Nursing	2221	93.8 (92.7–94.8)
Allied health	817	93.5 (91.6–95.1)
Ancillary	744	93.0 (90.9–94.7)
Doctor/dentist	124	94.4 (88.7–97.7)
Administrators	558	90.6 (88.0–93.0)
Service-provider	411	88.1 (84.5–91.0)

**Table 4:** Crude seroprevalence by nationality in 2014 (n = 4875)

Nationality	n	Seroprevalence in % (binomial 95 % CI)
Singapore	2660	93.2 (92.1–94.1)
Philippines	776	92.9 (90.8–94.6)
Malaysia	418	94.7 (92.1–96.7)
China	257	98.4 (96.0–99.6)
India	160	82.5 (75.7–88.0)
Myanmar	107	96.3 (90.7–99.0)
Other	86	91.9 (83.9–96.7)
Missing	411	88.1 (84.5–91.0)

We conducted a multiple logistic regression analysis on the immunity status (Table 5). The year of sampling was included in the final model in order to adjust for the confounding effect of a change in policy. There was no significant difference in odds of being immune between male and female employees. The adjusted odds of being immune were significantly lower for employees born in more recent decades. Employees of Indian nationality were significantly less likely to be immune than their Singaporean co-workers while Chinese nationals had higher odds of immunity. Those working in administrative areas or as service-providers were significantly less likely to be seropositive than employees in the nursing vocation.

**Table 5:** Multiple logistic regression on final immune status in 2014 (n = 4875)

Exposure variable	Odds ratio	*p* value	95 % CI	
			Lower	Upper
Year				
2009	1.00	–	–	–
2011	1.95	0.051	0.998	3.82
2012	0.927	0.775	0.552	1.56
2014	2.51	<0.001	1.53	4.11
Gender				
Female	1.00	–	–	–
Male	0.797	0.158	0.582	1.09
Age-group by year of birth				
≤1959	1.00	–	–	–
1960–1969	0.563	0.052	0.316	1.00
1970–1979	0.313	<0.001	0.187	0.525
≥1980	0.209	<0.001	0.126	0.348
Vocation				
Nursing	1.00	–	–	–
Allied health	0.896	0.545	0.628	1.28
Ancillary	0.591	0.006	0.404	0.862
Doctor/dentist	1.02	0.959	0.441	2.37
Admin	0.435	<0.001	0.294	0.642
Service-provider	0.302	<0.001	0.193	0.473
Nationality				
Singapore	1.00	–	–	–
Philippines	0.806	0.239	0.563	1.15
Malaysia	1.29	0.287	0.808	2.05
China	4.43	0.004	1.61	12.2
India	0.268	<0.001	0.166	0.433
Myanmar	1.63	0.351	0.584	4.55
Others	0.820	0.634	0.362	1.86
Missing^a^	1.00	–	–	–

^a^Missing data on nationality was perfectly collinear with service-provider vocation

## Discussion

We have reported the estimated seroprevalence rates of a tertiary hospital in Singapore from 2009 to 2014, and we have found significantly higher immunity 1 year after instituting a mandatory varicella seroprotection policy. However, we still have a small proportion of individuals who are not immune to varicella at least by serological screening. Considering how our methods did not include follow-up testing for ‘borderline’ titres, it is likely that we have overestimated the sensitivity and specificity of our serological test. We could therefore infer that the true proportion of non-immune individuals may in fact be larger than estimated. Multiple logistic regression analysis showed lower immunity among employees from India and those working in non-clinical vocations.

Our crude annual seroprevalence estimates were in keeping with reported national rates for adults which ranged between 84.0 % (ages 18–29) and 96.4 % (ages 70–79) [3]. The logistic regression performed on the 2014 data showed that staff born in the 1970s and 1980s were at significantly lower odds of being immune than those born in the 1950s, even after controlling for sex, nationality and vocation.

One paediatric hospital in Singapore had previously estimated that between 92.3 and 93.5 % of its healthcare workers (HCW) were immune to VZV, using self-reported history of infection and objective serum samples where employees were unsure of their history [8]. One hospital in Taiwan reported a laboratory-confirmed seroprevalance rate of 91.1 % among its HCWs [9]. Our consolidated hospital-wide seroprevalence of 92.8 % (95 % CI 92.1–93.5) in 2014 was comparable. Still, we were not able to achieve the rates reported in Japanese healthcare institutions which can range between 94.7 and 97.4 % [10–12]. Data on HCWs in the wider Southeast Asian region are not available for comparison, save for a small sample studied in Malaysia, which showed a seroprevalence of 84.4 % [13].

In this audit we also described a multi-national workforce which comprised 51 % Singaporean citizens and 49 % foreign nationals, drawn predominantly from Southeast, East and South Asia. We were able to draw only limited comparisons with multi-national HCW data from Saudi Arabia [14] who reported an 81 % seroprevalence among HCWs from the “Far East”.

Our multiple logistic regression showed that the odds of being immune was higher for People’s Republic of China (PRC) nationals than for Singaporeans. While we were unable to find data on HCWs from the PRC, we noted that pregnant women in Hong Kong [15], who are likely of similar ages compared to our HCWs, were shown to have a seroprevalence as high as 95.4 %.

The differences in immunity seen between nationalities might be explained by past observations how a tropical climate affects the transmission patterns of varicella, allowing the disease to behave more like a disease of early adulthood [16, 17]. This would explain higher immunity in employees from the PRC, a predominantly temperate country, compared to the rates seen in Singapore, Malaysia, India, the Philippines and Myanmar, countries located within the tropics. The reasons for this geographical distribution of sero-epidemiology have never been completely explained.

We were especially interested to see that Indian nationality in our HCW population was associated with a lower odds of being seropositive. We found literature reporting seroprevalence rates between 88 and 91 % in the young adult population in India [18]. Data on Singaporean military recruits reflected that ethnic Indians had a lower seroprevalence rate than ethnic Chinese or Malays [19]. This finding, however, was not reproduced in national health survey data [3]. We would infer that varicella immunity was less likely a function of ethnicity, more likely a result of birthplace or nationality. Again, the reasons for a lower seroprevalence among those born in India working in Singapore has not been fully explained.

Past policy had made provisions for HCWs to be offered free vaccination in conjunction with outbreak investigations and annual screening exercises. The significant increase in seroprevalence recorded in 2014 coincided with a change in pre-employment policy 1 year prior, which required new employees to be serologically screened and vaccinated. Those found to be non-immune received two doses of vaccine. Sero-conversion data were not collected.

The fact that administrative staff and service providers were less likely to be immune than their nursing colleagues is in keeping with past vaccination policies which favoured clinicians and the higher likelihood of clinical staff being exposed through their line of work. Nonetheless, given that varicella is an airborne infectious disease [20] which does not discriminate between vocations, more could be done to ensure that non-clinical members of the workforce are vaccinated. Given a fairly consistent rate of staff turn-over, it remains to be seen whether the seroprevalence will continue to rise or plateau at the 2014 level.

Our findings and analysis were limited by the overall rates of participation in health screening; our consolidated data-set for 2014 failed to capture results for two-fifths of the hospital’s full-time staff. Doctors, male members of staff and younger individuals were over-represented in this group. Given that the purpose of annual health screening was chronic disease prevention, we had to acknowledge a selection bias for older participants. Given that younger staff members were less likely to have been immune, we had to concede that our seroprevalence figures may have represented an overestimate.

A post hoc review of missing data showed that human resource records lacked information on the nationalities of employees working for service-provider companies. We did not include vaccination status in our analysis as this information was not captured as part of the screening exercise. Observed hospital-wide trends might have been driven by changes in seroprevalence within non-clinical staff, whose vaccination may play a relatively smaller role in total patient safety. Nevertheless, some of our findings may help to inform vaccination policy across hospitals in Singapore and the wider Southeast Asian region.

## Conclusions

Having reviewed 5 years of data, it has become evident that certain sub-populations in our workforce might benefit from additional screening and vaccination measures. Future studies would need to include immunisation records to shed light on whether immunity has been acquired naturally or through vaccination. Despite a mandatory policy for new staff, we still have a residual population who are not immune to varicella. These individuals could pose a potential risk to themselves and to our patients who may be vulnerable to the complications of varicella. We have recorded improvements in seropositivity but more needs to be done to ensure that staff, patients and visitors in our hospitals are protected from this vaccine preventable infectious disease.

## Abbreviations

CI: confidence interval
ELISA: enzyme-linked immunosorbent assay
HCW: healthcare worker
OR: odds ratio
PRC: People’s Republic of China
STROBE: Strengthening the Reporting of Observational Studies in Epidemiology
VZV: varicella zoster virus
